# Genesis of placental sequestration in malaria and possible targets for drugs for placental malaria

**DOI:** 10.1002/bdr2.1496

**Published:** 2019-03-28

**Authors:** Robert L. Clark

**Affiliations:** ^1^ Artemis Pharmaceutical Research Jacksonville Florida

**Keywords:** fibrin, heparanase, malaria, metalloproteinase, placenta, sequestration, syndecan‐1

## Abstract

Malaria during pregnancy results in intrauterine growth restriction, fetal anemia, and infant mortality. Women are more susceptible to malaria during pregnancy due to malaria‐induced inflammation and the sequestration of infected red blood cells in the placenta, which bind to the chondroitin sulfate portion of syndecan‐1 on the syncytiotrophoblast and in the intervillous space. Syndecan‐1 is a dimeric proteoglycan with an extracellular ectodomain that is cleaved from the transmembrane domain (referred to as “shedding”) by matrix metalloproteinases (MMPs), likely the secreted MMP‐9. The ectodomain includes four binding sites for chondroitin sulfate, which are proximal to the transmembrane domain, and six distal binding sites primarily for heparan sulfate. This “shedding” of syndecan‐1 is inhibited by the presence of the heparan sulfate chains, which can be removed by heparanase. The intervillous space contains fibrin strands and syndecan‐1 ectodomains free of heparan sulfate. The following is proposed as the sequence of events that leads to and is primarily responsible for sequestration in the intervillous space of the placenta. Inflammation associated with malaria triggers increased heparanase activity that degrades the heparan sulfate on the membrane‐bound syndecan‐1. Inflammation also upregulates MMP‐9 and the removal of heparan sulfate gives MMP‐9 access to cleave syndecan‐1, thereby releasing dimeric syndecan‐1 ectodomains with at least four chondroitin sulfate chains attached. These multivalent ectodomains bind infected RBCs together leading to their aggregation and entrapment in intervillous fibrin. This mechanism suggests possible new targets for anti‐placental malaria drugs such as the inhibition of MMP‐9. Doxycycline is an antimalarial drug which inhibits MMP‐9.

**Abbreviations**
CSA: chondroitin sulfate A
CSC: chondroitin sulfate C
CSPG: chondroitin sulfate proteoglycan
DBL: Duffy binding like
kDa: kilodaltons
MMP: matrix metalloproteinase
MT‐MMPs: membrane‐type matrix metalloproteinase
PfEMP1: *Plasmodium falciparum* erythrocyte membrane protein 1
RBC: red blood cell

## 1 INTRODUCTION

Pregnant women are more susceptible to malaria and more severely affected (Beaudet et al., 2014; Huynh, Cottrell, Cot, & Briand, 2015; Kourtis, Read, & Jamieson, 2014; Menendez, 2006; Moore et al., 2017). This is particularly true for pregnant women infected with *Plasmodium falciparum* due to inflammation and cell sequestration in the placenta (referred to as placental malaria). *P. falciparum* causes the most severe disease and is the most deadly among several species of the unicellular protozoan genus *Plasmodium* (White et al., 2014). The next most important species is *Plasmodium vivax*, which causes less placental infection and no observed placental inflammation (Carter & Mendis, 2002; Chaikitgosiyakul et al., 2014; Mayor et al., 2012; McGready et al., 2004).

The virulence of *P. falciparum* is largely due to two features. First, *P. falciparum* parasites can infect all red blood cells (RBCs) whereas *P. vivax* infects only reticulocytes and relatively young erythrocytes (Douglas et al., 2012). Second, RBCs infected by certain stages (trophozoites and schizonts) of *P. falciparum* can adhere to the endothelial walls of blood vessels, other RBCs, and platelets leading to extensive sequestration in the microvasculature (Franke‐Fayard, Fonager, Braks, Khan, & Janse, 2010; Haldar, Murphy, Milner, & Taylor, 2007; Rowe, Claessens, Corrigan, & Arman, 2009; Udeinya, Miller, McGregor, & Jensen, 1983). This allows these parasites to evade the spleen where the infected RBCs could be removed from circulation and destroyed. Adhesion and sequestration occurs with *P. vivax* (Costa et al., 2011) but to a lesser extent than *P. falciparum*. In areas where *P. falciparum* malaria is endemic, the most sensitive population is children who have not yet developed immunity to malaria. The next most sensitive population is pregnant women, particularly primigravida and secundigravida, who may have immunity to common malaria (to be referred to as nonplacental malaria or peripheral malaria) but not to the pronounced sequestration of infected RBCs that occur in placental malaria.

### 1.1 The asexual *P. falciparum* life cycle and inflammation

Humans get malaria when they receive a bite from an infected female mosquito of the genus *Anopheles* (Michalakis & Renaud, 2009). Parasites infect the salivary glands of the mosquito and the malarial sporozoites are carried into the host blood in the saliva from the mosquito (Beier, 1998). Sporozoites infect hepatocytes in the liver where, over a period of 8–10 days, they develop into schizonts that each can release about a thousand merozoites into the blood. Merozoites rapidly invade RBCs where the parasites feed on hemoglobin which is plentiful in infected red cells. Free heme is generated and detoxified in the parasite's digestive vacuole by converting it into inert dark brown crystals referred to as malaria pigment or hemozoin. Merozoites develop into the ring stage which then go on to develop into trophozoites and then schizonts. Infected RBCs containing schizonts then burst and release more merozoites into the blood (Cowman & Crabb, 2006). RBCs infected with trophozoites and schizonts are the ones capable of adhering to the endothelium and thus the form of the parasite most commonly observed in a peripheral blood smear is the ring form which infects RBCs that do not adhere to the endothelium.

When the schizonts burst to release new merozoites, they also release hemozoin, cytokines, and other toxic factors. The hemozoin and cytokines are pro‐inflammatory and attract leukocytes to the sites of sequestration (Ordi et al., 1998; Pichyangkul, Saengkrai, & Webster, 1994; Prato, Giribaldi, Polimeni, Gallo, & Arese, 2005; Sherry et al., 1995). The resulting inflammation is responsible for the characteristic symptoms of severe malaria such as fever, chills, and impaired consciousness (Clark, Budd, Alleva, & Cowden, 2006; Cowman & Crabb, 2006; Rasti, Wahlgren, & Chen, 2004; Vogetseder, Ospelt, Reindl, Schober, & Schmutzhard, 2004).

### 1.2 Mechanism of adhesion and sequestration in nonplacental malaria

The *P. falciparum* parasites generate protein receptors, referred to as *P. falciparum* erythrocyte membrane protein 1 (PfEMP1) receptors, that become embedded in the plasma membrane of the RBC (Gamain, Gratepanche, Miller, & Baruch, 2002; Rowe et al., 2009). These receptors form clusters on the cell surface, which are referred to as knobs (Duffy & Fried, 1999; Udeinya et al., 1983). Different forms of the PfEMP1 receptors can bind to a variety of targets (including endothelial surface molecules such as CD36, ICAM1, thrombospondin, VCAM‐1, E‐selectin, P‐selectin, and CD31; Rowe et al., 2009) but each infected RBC commonly binds to one type of target receptor. In general, sequestration in the microvasculature results from the binding of the cysteine‐rich interdomain region 1 of PfEMP1 receptors to the CD36 glycoprotein receptors on the surface of endothelium, RBCs, infected RBCs, and platelets (Gamain et al., 2002; Rowe et al., 2009). The binding selectivity of the PfEMP1 receptor can be switched at each new asexual cycle allowing the parasite to adapt to new conditions.

The sequestration leads to microcirculatory obstruction which causes impaired tissue perfusion leading to local hypoxia (Clark et al., 2006). Infected RBCs are better at adhering to the vascular endothelium of certain tissues including the bone marrow (Ekvall, 2003; Haldar & Mohandas, 2009; Wickramasinghe & Abdalla, 2000; Wickramasinghe, Phillips, Looareesuwan, Warrell, & Hughes, 1987). This adhesion in the bone marrow impacts RBC production which contributes to anemia (Franke‐Fayard et al., 2010; Haldar & Mohandas, 2009; Lamikanra et al., 2007; Obaldia et al., 2018; Smalley, Abdalla, & Brown, 1980). With successive malarial infections, the host becomes able to generate antibodies that block the binding of the PfEMP1 receptors to the CD36 glycoprotein receptors (Doolan, Dobaño, & Baird, 2009). In endemic areas, most adults have acquired this immunity.

## 2 MALARIA IN PREGNANCY

During pregnancy, women in endemic areas become more susceptible to malaria infection and are more severely affected. This sensitivity is due to the generation of infected RBCs with PfEMP1 receptors with active Duffy binding‐like (DBL)γ domains (referred to as VAR2CSA receptors) that can bind to the chondroitin sulfate A (CSA) portion of chondroitin sulfate A proteoglycans which is the target receptor in the placenta. These infected RBCs with VAR2CSA receptors start out as a small portion of the total infected RBCS but are selected for and develop rapidly (Achur, Valiyaveettil, Alkhalil, Ockenhouse, & Gowda, 2000; Brabin et al., 2004; Salanti et al., 2004; Srivastava et al., 2010; Ezebialu et al., 2012; Ouédraogo et al., 2012; Kalilani‐Phiri et al., 2013; Pehrson et al., 2016; Sharma & Shukla, 2017). Women in endemic areas likely have immunity to the common form of malaria caused by infected RBCs that bind to CD36 receptors but primigravidae do not have immunity to VAR2CSA‐binding infected RBCs that bind in the placenta and cause placental malaria. Infected pregnant women eventually form antibodies that block the binding of infected RBCs to chondroitin sulfate A proteoglycans and infections during subsequent pregnancies are not as severe (Desai et al., 2007; Duffy & Fried, 2003; Ezebialu et al., 2012; McLean, Ataide, Simpson, Beeson, & Fowkes, 2015; Moore et al., 2017; Rogerson, 2017; Rogerson, Hviid, Duffy, Leke, & Taylor, 2007; Sharma & Shukla, 2017; Uneke, 2007a, 2007b).

The prevalence of malaria in pregnancy depends on the stage of gestation. For example, in one study, the prevalence of peripheral malaria for primigravidae in Kenya peaked at 85.7% between gestation Weeks 13 and 16 and then gradually decreased to 42.9% between gestation Weeks 33 and 36 (Brabin, 1983). The pattern for multigravidae was similar but lower with a peak of 51.7% between gestation Weeks 13 and 16 and 23.5% between Weeks 33 and 36. In an area of low transmission (the Thailand–Myanmar border), a total of 16.4% (8221) of 50,060 pregnant women had *falciparum* and/or *vivax* malaria (6.3% with *falciparum* malaria only, 7.2% with *vivax* malaria only and 3.0% with both; Moore et al., 2017). In this study, the peak rates of malaria were observed at gestation Weeks 6 and 5 for *P. falciparum* and *P. vivax*, respectively.

## 3 CHARACTERISTICS OF PLACENTAL MALARIA

Infected RBCs with VAR2CSA receptors are found attached to the apical lining of the syncytiotrophoblast but even more notably in the intervillous space, sometimes together with monocytes and macrophages with ingested pigment (Beeson, Amin, Kanjala, & Rogerson, 2002; Brabin, 1983; Chaikitgosiyakul et al., 2014; de Moraes, Tadokoro, Gómez‐Conde, Olivieri, & Penha‐Gonçalves, 2013; Duffy & Fried, 2001a, 2001b; Fried & Duffy, 1996; Imamura, Sugiyama, Cuevas, Makunde, & Nakamura, 2002; Ismail et al., 2000; Muthusamy et al., 2004; Walter, Garin, & Blot, 1982). This congestion leads to decreased uterine artery blood flow (Brabin & Johnson, 2005; Dorman et al., 2002; Griffin et al., 2012; Umbers, Aitken, & Rogerson, 2011). Patients with placental malaria may or may not also have an apparent peripheral blood infection. In one study, among 109 patients with acute placental malarial, peripheral blood malaria was apparent by microscopy in 47% of cases (Ismail et al., 2000). In another study, 254 (70%) of 365 women with peripheral malaria also had parasitized term placentas resulting in a significant (*p* < 0.001) association between peripheral malaria and placental malaria (Ezebialu et al., 2012).

### 3.1 Prevalence of placental malaria

As would be expected, placental malaria is more common in areas where malaria is endemic (Fehintola et al., 2017). In sub‐Saharan Africa, placental malaria is almost entirely due to *P. falciparum*. Across 21 studies conducted in nine sub‐Saharan African countries in which evidence of malaria was not a criterion for enrollment (studies were reported between 1925 and 2017), the percentage of 10,032 term pregnancies that were infected with placental malaria was 29.9% (Adebami, Owa, Oyedeji, Oyelami, & Omoniyi‐Esan, 2007; Anagnos, Lanoie, Palmieri, Ziefer, & Connor, 1986; Bassey, Nyengidiki, & John, 2015; Brabin, 1983; Bulmer, Rasheed, Francis, Morrison, & Greenwood, 1993; Ezebialu et al., 2012; Fehintola et al., 2017; Kalilani‐Phiri et al., 2013; Matteelli et al., 1994; Menendez et al., 2000). In areas of low transmission, the rate of placental malaria is lower. Among 400 pregnant women in a study conducted in Panama (Clark, 1915), there were 19 with placental malaria (4.8%).

### 3.2 Relationship between placental malaria and stage of gestation

In most studies, the definitive diagnosis of placental malaria has been based on the histological examination of the term placenta and, in general, there is little information about the extent of placental malaria early in gestation (Cohee et al., 2016; Valea et al., 2012). Reports of the stages of pregnancy that can be affected include women who are earlier than 4 months of pregnancy (Cottrell, Mary, Barro, & Cot, 2007), earlier than 20 weeks of gestation (Griffin et al., 2012), and 10 weeks of gestation and greater (Valea et al., 2012). In another study, 46 of 75 (48%) women had parasitized placentas following peripheral malaria detected during the second trimester and 10 of 75 (13%) had parasitized placentas following peripheral malaria detected during the third trimester (Kalilani‐Phiri et al., 2013).

Other studies, though, have used methods to detect the presence of placental infected RBCs in peripheral blood prior to delivery. For example, isolates of infected RBCs collected from peripheral blood can be evaluated for transcripts of the *var2csa* gene by polymerase chain reaction (PCR; Snounou et al., 1993; Mockenhaupt, Ulmen, von Gaertner, Bedu‐Addo, & Bienzle, 2002). In a study in Benin, pregnant women were enrolled during the first or second trimester. At enrollment, the median gestational age was 15.9 weeks and the interquartile range was 13.7–17.0 weeks. Peripheral blood was collected periodically resulting in 50 samples collected prior to delivery and 41 samples at delivery (Doritchamou et al., 2012). For analysis, the samples were placed into three categories based on when they were collected—prior to gestation week 13, after gestation week 13 but before delivery, and at delivery. A variety of approaches including the evaluation of *var2csa* gene transcripts using PCR was used to show that some of the women from all stages had infected RBCs in peripheral blood with the placental phenotype (i.e., binding to chondroitin sulfate A) and that the affinity of the infected RBCs for chondroitin sulfate was similar for the three stages of gestation evaluated.

### 3.3 Stages of infection in the placenta

The stages of infection within the placenta have been identified histologically and one system of classification originally developed by Bulmer, Rasheed, Francis, et al. (1993), Bulmer, Rasheed, Morrison, Francis, and Greenwood (1993), and subsequently followed by Adebami et al. (2007) and Ezebialu et al. (2012), is shown below:

- Active infection: Parasites and pigments in maternal RBCs in the intervillous spaces but no pigment or cells within fibrin.
- Active‐on‐past infection: Parasites and pigment in maternal RBCs and pigment and/or cells within fibrin.
- Past infection: Parasites not present and pigment and/or cells confined to fibrin.

In Bulmer, Rasheed, Francis, et al. (1993), 121 placentas from women in The Gambia were examined and 9.9% had active infections, 16.5% had active‐on‐past infections, and 29.8% showed evidence of past infections. In Ezebialu et al. (2012), 365 placentas from women in Nigeria were examined, 6.3% showed active infection, 53.7% showed active‐on‐past infections, and 9.6% showed past infection. Note: in Bulmer, Rasheed, Francis, et al. (1993), fibrin is described as both intervillous fibrin and perivillous fibrin although detailed definitions are not provided. The terminology and definitions to be used below are as follows. The term intervillous fibrin will refer to the phenomenon wherein fibrin strands are observed across the intervillous space of the placenta and are not limited to the space close to the villi. Dense deposits of fibrin that occur on the surface of the villi will be referred to as perivillous fibrinoids (after Frank et al. (1994) and Kaufmann, Huppertz, and Frank (1996)).

### 3.4 Other factors

In addition to sequestration in the placenta, the severity of the disease and the outcome of the pregnancy in women with malaria are adversely impacted by the following factors:

- Maternal anemia (Brabin, 1983; Matteelli et al., 1994; Guyatt & Snow, 2001; Uneke, 2007a, 2007b; Mokuolu et al., 2009; Prentice et al., 2010; Verhoef et al., 2010; Kalilani‐Phiri et al., 2013; Bassey et al., 2015; Maternal and Child Survival Program (MCSP), President's Malaria Initiative (PMI), and Centers for Disease Control (CDC), 2015; Fehintola et al., 2017);
- Pre‐eclampsia (Brabin & Johnson, 2005), and,
- Intervillous infiltration of mononuclear inflammatory cells and macrophages (Carmona‐Fonseca, Arango, & Maestre, 2013; Imamura et al., 2002; Ismail et al., 2000; Maeno et al., 1993; Muehlenbachs et al., 2010; Ordi et al., 1998; Sharma & Shukla, 2017; Uneke, 2007a) that are associated with the malaria‐induced release of chemokines and cytokines in the placenta (Fried et al., 1998; Suguitan et al., 2003).

## 4 IMPACT OF MALARIA ON THE OFFSPRING

The potential impact of malaria on the offspring is reviewed in Menendez et al. (2000), Dorman et al. (2002), Uneke (2007a), (2007b), and De Beaudrap et al. (2013). One common effect is intrauterine growth restriction and low birth weight (Adebami et al., 2007; Bassey et al., 2015; Brabin et al., 2004; Chandrasiri et al., 2014; Cottrell et al., 2007; de Moraes et al., 2013; Gaccioli & Lager, 2016; Griffin et al., 2012; Huynh et al., 2015; Matteelli, Caligaris, Castelli, & Carosi, 1997; Mokuolu et al., 2009; Moore et al., 2017; Rasti et al., 2004; Rijken et al., 2012; Umbers et al., 2011). In a study in Burkina Faso (Valea et al., 2012), women first infected in the first trimester were more likely to have babies with low birth weight (incidence 13/31 = 42%) than women first infected in the second and third semesters. In a study of 832 infants in Uganda (De Beaudrap et al., 2016), postnatal growth was most affected if the mother had had malaria during the last 12 weeks of pregnancy. Malaria in pregnancy is also associated with prenatal, perinatal, and postnatal mortality (McGready et al., 2012; Schantz‐Dunn & Nour, 2009) and it has been estimated that 75,000–200,000 infant deaths each year are attributable to malaria in pregnancy (Steketee, Nahlen, Parise, & Menendez, 2001).

Other potential adverse effects on the offspring are fetal anemia (Brabin et al., 2004; le Cessie et al., 2002; Moore et al., 2017; Uneke, 2007b), postnatal anemia (Accrombessi et al., 2015), preterm birth (De Beaudrap et al., 2016; Dorman et al., 2002; Menendez et al., 2000; Schantz‐Dunn & Nour, 2009), and transplacental transmission of malaria/congenital malaria (Bergström et al., 1993; De Beaudrap et al., 2016).

## 5 ROLE OF CHONDROITIN SULFATE PROTEOGLYCANS IN PLACENTAL MALARIA

### 5.1 Glycosaminoglycans and proteoglycans in humans

The endothelial glycocalyx consists of a network of membrane‐bound proteoglycans and glycoproteins which cover the endothelium on the luminal side (Pries, Secomb, & Gaehtgens, 2000; Reitsma, Slaaf, Vink, van Zandvoort, & oude Egbrink, 2007). Proteoglycans consist of core proteins with attached glycosaminoglycans (GAGs). GAGs are unbranched chains of repeating disaccharide units and, with the exceptions of heparin and hyaluronic acid, are attached to core proteins. Proteoglycans can have from 1 to more than 100 GAG side chains, each attached to a serine side chain on the core protein via a specific tetrasaccharide link (Alberts et al., 2015). Also, the length of the GAG side chains varies. For most proteoglycans, the attached GAGs consist of chains of chondroitin sulfate and/or heparan sulfate (Alexopoulou, Multhaupt, & Couchman, 2007; Deepa et al., 2004; Elenius & Jalkanen, 1994; Kokenyesi & Bernfield, 1994). The sulfation of chondroitin and heparan occurs by chondroitin sulfate sulfotransferases and heparan sulfate transferases, respectively. Sulfate groups can be removed from some GAGs by extracellular endosulfatases (Morimoto‐Tomita, Uchimura, Werb, Hemmerich, & Rosen, 2002; Yang et al., 2007).

Chondroitin sulfate is actually the name of a family of GAGs. The disaccharide repeating units of chondroitin sulfate can consist of d‐glucuronic acid and *N*‐acetylgalactosamine with *N*‐acetylgalactosamine being sulfated in the 4‐carbon position (referred to as chondroitin‐4‐sulfate or chondroitin sulfate A [CSA]) or the 6‐carbon position (referred to as chondroitin‐6‐sulfate or chondroitin sulfate C [CSC]). In some cases, both chondroitin sulfate A disaccharides and chondroitin sulfate C disaccharides can be included within the same chain. Alternatively, the disaccharide can consist of l‐iduronic acid (instead of glucuronic acid) and galactosamine (instead of *N*‐acetylgalactosamine) with the 4‐carbon position of the galactosamine being sulfated (referred to as chondroitin sulfate B or dermatan sulfate). Chondroitin sulfate proteoglycans (CSPGs) are cell surface and extracellular matrix components.

### 5.2 Evidence that the ligand for placental malaria is chondroitin sulfate A

Various lines of evidence suggest that infected RBCs with VAR2CSA receptors bind specifically to the chondroitin sulfate A portion of chondroitin sulfate A proteoglycans on the surface of the syncytiotrophoblast and in the intervillous space of the placenta. There is also extensive evidence that these infected RBCs with VAR2CSA receptors that bind to chondroitin sulfate A are responsible for the sequestration of cells in the placenta. First, Rogerson, Chaiyaroj, Ng, Reeder, and Brown (1995) showed that RBCs infected with certain strains of *P. falciparum* can bind to chondroitin sulfate A attached to a plastic Petri dish. Fried and Duffy (1996) showed that:

- Infected RBCs from term infected placentas would bind to chondroitin sulfate A attached to a plastic Petri dish but not to other extracellular matrix proteins or to other known IRBC receptors, whereas infected RBCs from nonpregnant donors did not bind to chondroitin sulfate A.
- Infected RBCs from infected placentas would also bind within the intervillous space in sections from uninfected term placentas and this binding was inhibited by chondroitin sulfate A.
- Peripheral infected RBCs from nonpregnant women bound to CD36 attached to a plastic Petri dish but not to chondroitin sulfate A. Peripheral infected RBCs from pregnant women could bind to either CD36 or chondroitin sulfate A.

Gysin, Pouvelle, Fievet, Scherf, and Lépolard (1999) showed that soluble chondroitin sulfate A can break apart the clusters of cells involved in placental malaria.

These findings were confirmed and extended in multiple subsequent studies (Achur et al., 2000; Beeson, Chai, Rogerson, Lawson, & Brown, 1998; Cooke, Rogerson, Brown, & Coppel, 1996; Duffy & Fried, 1999; Fried & Duffy, 1998; Fried, Lauder, & Duffy, 2000; Pouvelle, Fusaï, Lépolard, & Gysin, 1998). More recently, it was shown in an ex vivo model of perfused placental tissue that soluble chondroitin sulfate A and specific antibodies directed against VAR2CSA inhibited the binding of infected RBCs (Pehrson et al., 2016). Also, it was found that soluble chondroitin sulfate A inhibited the binding of the recombinant VAR2CSA receptor to both the syncytiotrophoblast and the intervillous mesh in tissue sections of term placentas (Ayres Pereira et al., 2016). The same authors reported that an antibody to chondroitin sulfate (CS‐56), which binds to both chondroitin sulfate A and chondroitin sulfate C, bound in placental tissue sections to both the syncytiotrophoblast and the intervillous mesh with a pattern similar to that of VAR2CSA as well as the truncated active portion of recombinant VAR2CSA previously reported on by Clausen et al. (2012) (referred to in Ayres Pereira et al. (2016), as rVAR2).

### 5.3 Characterization of the VAR2CSA receptor for chondroitin sulfate A

Clausen et al., 2012 studied the properties of different fragments of the PfEMP1 receptor VAR2CSA including the binding to isolated placental CSPG samples. They identified a minimal chondroitin sulfate A binding region (ID1‐DBL2Xb) with a molecular weight (MW) of 62 kDa that retained high affinity binding (*K*_D_ = 21.8 nM) to a CSPG, decorin, which is a small proteoglycan (MW ~40 kDa) with a single chondroitin sulfate chain. Larger VAR2CSA fragments which contained this 62 kDa segment had somewhat greater affinity including FV2 (the full‐length VAR2CSA protein without the N‐terminal segment) for which the *K*_D_ was 5.2 nM.

### 5.4 The size of the binding site for VAR2CSA on chondroitin sulfate A

Several studies have contributed to the characterization of the binding site(s) on the chondroitin sulfate A ligand. Both Beeson et al. (1998) and Pouvelle et al. (1998) prepared chondroitin sulfate A chains of various lengths and studied their effects on the binding of infected RBCs to chondroitin sulfate A attached to plastic Petri dishes. Beeson et al. observed that 90 μg/mL of an oligosaccharide with seven chondroitin sulfate A disaccharide units reduced infected RBC binding to 50% of control whereas 90 μg/mL of an oligosaccharide with eight chondroitin sulfate A disaccharide units reduced infected RBC binding to 20% of control. The same concentration of an oligosaccharide with seven chondroitin sulfate C disaccharide units did not inhibit infected RBC adhesion. Pouvelle et al. observed that 100 μg/mL of a chain of approximately 4 kDa (~9 chondroitin sulfate A disaccharide units) was needed to inhibit the binding of infected RBCs to chondroitin sulfate A by 50% and a chain of approximately 9 kDa (~19 disaccharide units) was needed to inhibit the binding by the same amount as the full VAR2CSA molecule.

Alkhalil, Achur, Valiyaveettil, Ockenhouse, and Gowda (2000) measured the adhesion of chondroitin sulfate A‐adherent infected RBCs to purified CSPGs from human placental intervillous space. They prepared oligosaccharides of chondroitin sulfate A of various sizes and with varying extent of sulfation and measured the ability of these oligosaccharides to inhibit the adhesion of the infected RBCs to chondroitin sulfate A. They reported that the maximal inhibition of infected RBC binding (90–93% inhibition) was seen with oligosaccharides consisting of six chondroitin disaccharide units and about 30–38% of maximal 4‐sulfate content. Oligosaccharides consisting of six chondroitin sulphate C disaccharide units and 89–98% of maximal sulfate content caused a lesser (32–36%) inhibition of infected RBC binding. Oligosaccharides consisting of six chondroitin sulfate C disaccharide units had no binding inhibitory activity. Chai, Beeson, and Lawson (2002) observed that the minimum chain length for effective inhibition of infected RBC binding consisted of six chondroitin disaccharide units with four or five being chondroitin sulfate A and one or two being unsulfated.

Thus, it seems likely that the binding site for VAR2CSA on placental chondroitin sulfate A consists of between 6 and 19 chondroitin disaccharide units.

### 5.5 Placental proteoglycans

#### 5.5.1 Distribution and characterization of chondroitin sulfate and heparan sulfate in the term human placenta

Achur et al. (2000) studied CSPGs and heparan sulfate from the uninfected term human placenta and the binding of infected RBCs to these CSPGs. The CSPGs that they identified were described as two major low‐sulfate CSPGs from the intervillous space, minor amounts of two different CSPGs associated with cells, and major amounts of dermatan sulfate‐like CSPG from the fibrous tissue. In the more common CSPG from the intervillous space, about one‐half of the *N*‐acetylgalactosamine groups in the GAG chains were sulfated in the 4‐position. In the less common CSPG from the intervillous space, about 1 in 10 of the *N*‐acetylgalactosamine groups in the GAG chains were sulfated in the 4‐position. There were also two primary CSPGs identified as being cell associated and these were also not fully sulfated with about one‐half of the *N*‐acetylgalactosamine groups being sulfated.

Achur et al., 2000 detected no heparan sulfate in the material from the intervillous space, whereas approximately 60% of the GAGs from the cell‐associated proteoglycans were degraded by heparinase III, indicating a large content of heparan sulfate.

Achur et al. (2000) also observed high affinity binding of infected RBCs to both the intervillous space‐derived CSPG and cell‐associated CSPG with greater affinity observed for the intervillous space‐derived CSPG. For both types of CSPGs, the binding was inhibited by soluble chondroitin sulfate A and prevented by pretreating the CSPGs with chondroitinase ABC which degrades chondroitin sulfate A, chondroitin sulfate B, and chondroitin sulfate C.

Beaudet et al. (2014) washed excess blood from three whole human placentas using chilled phosphate buffered saline and then dissected them into three regions—the cotyledons, the chorionic plate, and umbilical cord. The GAGs of each of the three regions were characterized. In the cotyledons (which presumably included much of the contents of the intervillous space), the GAGs included not only chondroitin sulfate A and unsulfated disaccharides but also chondroitin sulfate 6 and heparan sulfate. Among the three placentas, the ratio of chondroitin sulfate to heparan sulfate in the cotyledons ranged from 3 to 5. Achur et al. (2000) had not detected the presence of chondroitin sulfate C (C6S) in placental CSPG. Beaudet et al. had the following explanation: “The observation of 6S disaccharide and other minor disaccharides in the current study may be attributable to the inclusion of GAGs from the fibrous scaffold of the lobes and/or the increased sensitivity of the ultraperformance liquid chromatography‐mass spectrometric method used.”

Beaudet et al. also evaluated the binding of the GAG preparations from the three regions to the 62‐kDa chondroitin sulfate A binding fragment from VAR2CSA (ID1‐DBL2Xb) studied by Clausen et al. (2012). The affinity for the binding to the chondroitin sulfate A‐binding region (ID1‐DBL2Xb) by chondroitin sulfate isolated from the cotyledons was only slight less (*K*_D_ = 37 nM) than that obtained for the binding to the chondroitin sulfate A‐binding region (ID1‐DBL2Xb) by the CSPG decorin (*K*_D_ = 21.8 nM).

#### 5.5.2 Syndecan‐1

One of the proteoglycans of the endothelial glycocalyx of the human syncytiotrophoblast is syndecan‐1 (Jokimaa et al., 1998; Szabo et al., 2013). Syndecans are proteoglycan components of both the cell surface and the extracellular matrix. Syndecan‐1 exists as a dimer and each monomeric polypeptide has 311 amino acid residues, a molecular weight of 33 kDa and includes a short cytoplasmic domain of 34 amino acid residues, a single transmembrane domain of ~42 amino acid residues, and an extracellular ectodomain of ~235 amino acid residues (Elenius & Jalkanen, 1994; Kokenyesi & Bernfield, 1994; Manon‐Jensen, Itoh, & Couchman, 2010; Saunders, Jalkanen, O'Farrel, & Bernfield, 1989). Each syndecan‐1 monomer has five serine–glycine sites that serve as the attachment points for glycosylation. There are two sites on the end of the ectodomain proximal to the transmembrane domain that bind only chondroitin sulfate chains and three sites at the distal end of the ectodomain that primarily bind heparan sulfate but can also bind short chondroitin sulfate chains (Kokenyesi & Bernfield, 1994; Manon‐Jensen et al., 2010; Saunders et al., 1989). Depending on the source, the MW of syndecan‐1 monomers including the GAG chains range from 100 kDa to greater than 300 kDa (Ramani, Pruett, Thompson, DeLucas, & Sanderson, 2012).

Syndecan 1 is strongly expressed on the apical surface of syncytiotrophoblast cells (Jokimaa et al., 1998). The extracellular ectodomain can be cleaved from the transmembrane portion by matrix metalloproteinases (MMPs, discussed below) and become soluble (Bernfield et al., 1999). During gestation, the concentrations of syndecan‐1 (presumably ectodomain) in the serum from peripheral blood increased steadily at each time point (nonpregnant and gestational Weeks 10, 20, 30, and 38) through gestational Week 30 and, at gestational Week 38, there was a 159‐fold increase compared to nonpregnant controls (Hofmann‐Kiefer et al., 2013). In contrast, the serum concentrations of heparan sulfate remained fairly constant during gestation and, at gestational Week 38, represented only approximately 0.05% of the level of syndecan‐1.

Two findings suggest that the binding site for the VAR2CSA receptors on infected RBCs in the placenta is syndecan‐1 with chondroitin sulfate A chains (Ayres Pereira et al., 2016). First, syndecan‐1 was one of two CSPGs from placental extracts that bound to rVAR2 (the minimal active portion of recombinant VAR2CSA). Second, in a proximity ligation assay using tissue from third trimester placentas that had been perfused with RBCs infected with VAR2CSA‐expressing parasites, it was demonstrated that an antibody to syndecan‐1 colocalized with antibodies to VAR2CSA and rVAR2, binding to both the syncytiotrophoblast and the intervillous mesh.

Despite the extensive knowledge about the importance of syndecan‐1 and chondroitin sulfate in the sequestration of infected RBCs in the placenta, the precise mechanism for this accumulation of infected RBCs in placental malaria is not known.

#### 5.5.3 Metalloproteinases

The extracellular ectodomain of syndecan‐1 and other proteoglycans can be enzymatically cleaved close to the transmembrane domain by MMPs generating soluble proteoglycan ectodomains (referred to as “shedding”; Bernfield et al., 1999). MMPs are a family of zinc‐dependent endopeptidases that primarily act outside the cell and are involved in the breakdown of the extracellular matrix. MMPs are either secreted and soluble (Lichtenthaler et al., 2018), embedded in the plasma membrane (i.e., transmembrane) or outside the cell but anchored to a glycosylphosphatidylinositol group on the cell surface (Manon‐Jensen et al., 2010). The latter two types are referred to as membrane‐type MMPs (MT‐MMPs) and are primarily involved in pericellular degradation of the extracellular matrix whereas soluble MMPs are not constrained to the pericellular space (Itoh et al., 2001). Syndecan shedding by MMPs can be induced by several inflammatory factors associated with pathological conditions such as infection (Hayashida, Bartlett, Chen, & Park, 2010; Manon‐Jensen et al., 2010; Nam & Park, 2012; Park, Pier, Hinkes, & Bernfield, 2001; Teng, Aquino, & Park, 2012). The soluble syndecan‐1 ectodomain can compete with the intact membrane‐bound form for binding to extracellular ligands (Manon‐Jensen et al., 2010).

The MMPs that are both expressed in the syncytiotrophoblast (Chen & Khalil, 2017; Hiden et al., 2012; Kaitu'u‐Lino et al., 2012; Kaitu'u‐Lino, Palmer, Tuohey, Ye, & Tong, 2012; Majali‐Martinez et al., 2016; Sundrani, Chavan‐Gautam, Pisal, Mehendale, & Joshi, 2012) and capable of shedding syndecan‐1 (Brule et al., 2006; Endo et al., 2003; Fears & Woods, 2006; Hayashida et al., 2010; Li, Park, Wilson, & Parks, 2002; Purushothaman et al., 2010; Purushothaman, Chen, Yang, & Sanderson, 2008; Roper, Williamson, & Bass, 2012; Stepp, Pal‐Ghosh, Tadvalkar, & Pajoohesh‐Ganji, 2015; Vuoriluoto, Högnäs, Meller, Lehti, & Ivaska, 2011) are the secreted gelatinase MMP‐9 as well as MMP‐14 (MT1‐MMP) and MMP‐16 (MT3‐MMP) which are both transmembrane MMPs (Manon‐Jensen et al., 2010; Shofuda, Yasumitsu, & Nishihashi, 1997). The polypeptide structure of MMP‐9 includes three fibronectin II‐like repeats (Manon‐Jensen et al., 2010; Uhlin‐Hansen, 2013).

#### 5.5.4 Heparanase and MMP‐9

Using myeloma cells obtained from bone marrow aspirates (Yang et al., 2007) and multiple human cell lines (Ramani et al., 2012), it was demonstrated that the presence of heparan sulfate chains on syndecan‐1 inhibited the shedding of the syndecan‐1 ectodomain so a reduction in the heparan sulfate present on syndecan‐1 leads to increased shedding. Heparan sulfate chains on syndecan‐1 are degraded to oligosaccharides by heparanase which acts both at the cell surface and within the extracellular matrix and is upregulated by inflammation (Hulett et al., 1999; Ramani et al., 2012; Vlodavsky et al., 1999). The expression of heparanase in myeloma cell also led to the upregulation of MMP‐9, which sheds syndecan‐1 (Purushothaman et al., 2008). Thus, inflammation can induce a chain of events that leads to the increased shedding of the syndecan‐1 ectodomain.

MMP‐9 appears to have a role in inflammation and placental malaria. When human monocytes were incubated in vitro with *P. falciparum* trophozoite‐parasitized RBCs and hemozoin, the monocytes phagocytosed the infected RBCs and hemozoin, resulting in increased MMP‐9 activity and increased TNF‐α production (Prato et al., 2005). The secretion of MMP‐9 from cultured human microvascular endothelial cells was induced by exposing these cells in vitro to trophozoite‐parasitized RBCs (D'Alessandro, Basilico, & Prato, 2013). Other studies have also shown the possible pro‐inflammatory role of MMP‐9 in nonplacental malaria and some have suggested the potential use of MMP‐9 inhibitors as drugs for nonplacental malaria, particularly for complicated malaria (Dietmann et al., 2008; Polimeni & Prato, 2014; Prato & Giribaldi, 2011; Van den Steen et al., 2006). In a study in Ghana which compared 54 pregnant women with the minor T allele for an MMP‐9 promoter to 248 pregnant women with the more common CC genotype, the percent of women with peripheral parasites, placental parasites, and placental hemozoin was lower for the women with the T allele, suggesting a possible role for MMP‐9 in placental malaria (Apoorv et al., 2015).

## 6 ROLE OF FIBRIN IN THE ACCUMULATION OF INFECTED RBCS IN THE INTERVILLOUS SPACE

Intervillous fibrin was observed by light microscopy in the placentas of 20–29% of uninfected women in the United States who delivered early between gestational Weeks 23 and 27 (*N* ≥ 84 at each weekly gestational age; total *N* = 947; overall rate of intervillous fibrin was 24%; Hecht et al., 2008). In some extreme cases, fibrin can fill the intervillous space (Fuke et al., 1994; Waters & Ashikaga, 2006) and this observation has been associated with abortion (Gaither & Sampson, 1968).

It must be that intervillous fibrin is polymerized to be visible by light microscopy. The precursor to fibrin, fibrinogen, is a soluble glycoprotein (MW ~340 kDa) that is found in human plasma at concentrations normally in the range of 150–400 mg/dL (Asselta, Duga, & Tenchini, 2006; Cieśla, Adamczyk, Barbasz, & Wasilewska, 2013; Marucco, Fenoglio, Turci, & Fubini, 2013). Fibrinogen can bind to integrin α_IIb_β_3_ on the surface of platelets that have been appropriately activated (Du et al., 1993). Fibrinogen and fibrin also bind to fibronectin (Makogonenko, Tsurupa, Ingham, & Medved, 2002) and fibrin to MMP‐9 (Makowski and Ramsby, 1998). Thrombin is an enzyme that binds to the surface of circulating platelets and can convert fibrinogen to insoluble fibrin strands. This machinery is used for hemostasis which is normally prevented by factors released from the endothelial cells of intact blood vessels. However, the normal process of coagulation is disrupted by malaria (Angchaisuksiri, 2014; Vogetseder et al., 2004).

The initiation of the sequestration in the intervillous space in malaria has been described as free parasites becoming enmeshed in fibrin strands (Anagnos et al., 1986; Duffy & Fried, 2001a, p. 73). Extensive intervillous mesh‐like structures were detected by fibrin antibody in term placentas from women with malaria (Imamura et al., 2002). Muthusamy et al. (2004) describe fibrous, filamentous materials in the intervillous space of infected placentas. When infected RBCs from infected placentas were incubated with uninfected term placentas, the cells bound only in the periphery of the intervillous space (Fried & Duffy, 1996) indicating a difference been infected and uninfected placentas. However, in a subsequent study using placental‐type infected RBCs (i.e., expressing VAR2CSA), intervillous mesh was observed in the placentas from healthy women although the mesh was not as dense as seen with infected placentas (Ayres Pereira et al., 2016).

Fibrin can also contribute to the formation of perivillous or intravillous fibrinoids or perivillous fibrinoid plaques which occur on the surface of or within the placental villi of healthy women (Frank et al., 1994; Hargitai, Marton, & Cox, 2004; Kaufmann et al., 1996; Rampersad, Cervar‐Zivkovic, & Nelson, 2011). These fibrinoid deposits are a feature of placental malaria and, as mentioned above, when containing hemozoin or cells without parasites, are considered evidence of previous malaria infection (Bulmer, Rasheed, Francis, et al., 1993; Bulmer, Rasheed, Morrison, et al., 1993; Mostafa et al., 2015). When excessive, these perivillous fibrinoid deposits have been associated with a marked upregulation of platelet tissue factor that initiates thrombin formation from prothrombin. This upregulation of platelet tissue factor was suggested to be responsible for the formation of these malaria‐related fibrinoid deposits as a type of clotting (Imamura et al., 2002).

## 7 REVIEW OF KEY POINTS

### 7.1 In pregnant women with placental malaria

1. Placental malaria is caused by the sequestration of infected RBCs in the placenta and is associated with marked inflammation due partly at least to the release by infected RBCs of cytokines and hemozoin.
2. The binding of infected RBCs to the syncytiotrophoblast and in the intervillous space is caused by the binding of the PfEMP‐1 receptor VAR2CSA on the surface of infected RBCs to the chondroitin sulfate portion of a CSPG, likely syndecan‐1.

### 7.2 In various in vitro and in vivo human models

1. Syndecan‐1 is a transmembrane proteoglycan with an extracellular ectodomain and is found on the luminal surface of the syncytiotrophoblast.
2. The syndecan‐1 ectodomain is shed by MMP(s) and serum concentrations of syndecan‐1 ectodomain progressively increase during gestation.
3. Syndecan‐1 in the intervillous space must be the syndecan‐1 ectodomain as intact syndecan‐1 is a membrane protein.
4. Both the cell‐associated and intervillous proteoglycans have substantial chondroitin sulfate but there is substantial heparan sulfate only in the cell‐associated fraction.
5. The lack of heparan sulfate on the proteoglycans in the intervillous space could imply that the syndecan‐1 is not shed until the heparan sulfate on the syndecan‐1 ectodomain has been removed, presumably by heparanase.
6. Heparan sulfate on syndecan‐1 inhibits the shedding of the syndecan‐1 ectodomain, perhaps by forming a canopy that blocks the access of secreted MMPs to the base of the ectodomain.
7. Heparanase degrades the heparan sulfate on syndecan‐1 and is upregulated by inflammation.
8. Heparanase expression also leads to the upregulation of MMP‐9.
9. Inflammation is known to increase syndecan shedding and the upregulation of heparanase and MMP‐9 could be the primary mechanism.

## 8 PROPOSED MECHANISM FOR SEQUESTRATION IN THE INTERVILLOUS SPACE

**Figure 1:**
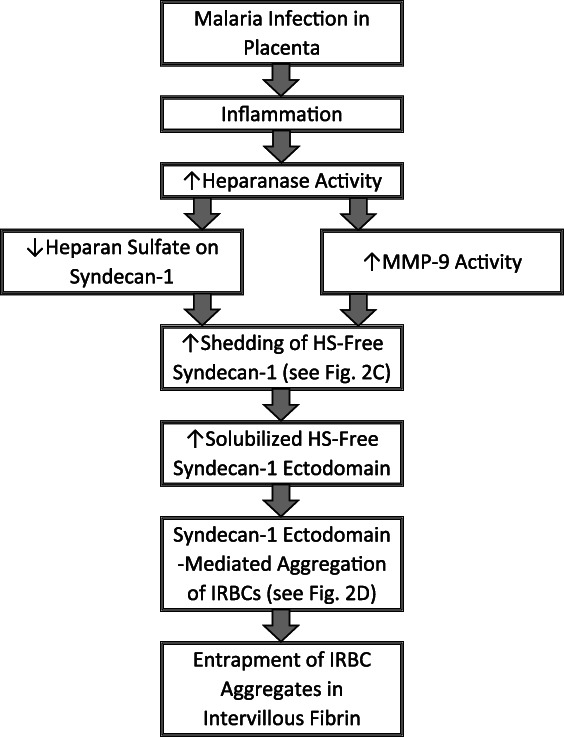
Flowchart of the proposed primary mechanism for placental malaria. Shown here are the steps outlined in Section 8. HS = heparan sulfate; infected RBCs = infected red blood cells; MMP‐9 = matrix metalloproteinase 9

**Figure 2:**
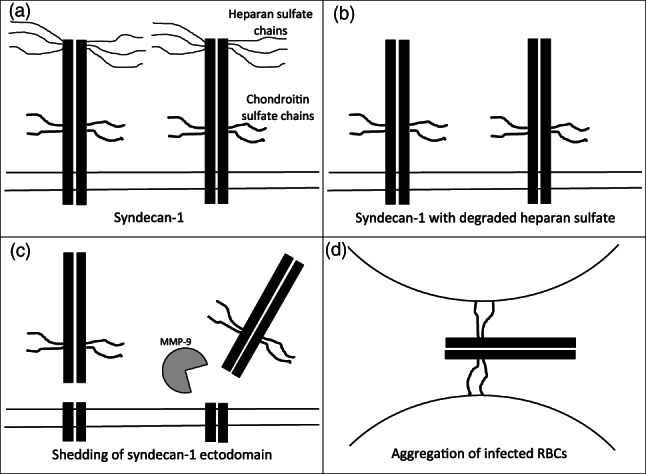
Schematic representation of the proposed sequential changes in syndecan‐1 in placental malaria. (a) A depiction of intact syndecan‐1 embedded in the plasma membrane of the syncytiotrophoblast showing the heparan sulfate chains on the ectodomain distal from the cell surface and the chondroitin sulfate chains on the ectodomain proximal to the cell surface. (b) Syndecan‐1 after the heparan sulfate chains have been removed by heparanase. (c) The removal of the heparan sulfate chains has cleared the way for MMP‐9 to access the ectodomain cleavage site near the surface of the syncytiotrophoblast. (d) The cleaved syndecan‐1 ectodomain with chondroitin sulfate chains has been released, allowing it to bind to multiple infected RBCs. The depictions of syndecan‐1 shown here were influenced by related models shown in Elenius and Jalkanen (1994), Bernfield et al. (1999), Pries et al. (2000), Manon‐Jensen et al. (2010), Roper et al. (2012), and Itoh et al. (2015). HS = heparan sulfate; infected RBCs = infected red blood cells; MMP‐9 = matrix metalloproteinase 9

The endothelial glycocalyx is complex. Nevertheless, it is proposed that the following cascade of events plays a significant role in the genesis of the sequestration of infected RBCs in the intervillous space in placental malaria (see Figures 1 and 2):

1. Since syndecan‐1 (see Figure 2a) appears not be shed until heparan sulfate has been removed, it is likely that syndecan‐1 is shed by a soluble MMP (i.e., MMP‐9) that would not have access to the ectodomain cleavage site until the canopy of heparan sulfate has been removed.
2. Inflammation associated with malaria triggers increased heparanase activity that degrades the heparan sulfate on the cell surface syndecan‐1.
3. The enhanced expression of heparanase then does both of the following:
  - It stimulates the formation of MMP‐9.
  - It removes the heparan sulfate barrier (Figure 2b) which enables the following:
    i Infected RBCs have access to and bind to the chondroitin sulfate chains on the intact membranous syndecan‐1 on the surface of the syncytiotrophoblast.
    ii Soluble MMP‐9 has access to the cleavage site at the base of the syndecan‐1 ectodomain.
4. These actions lead to the shedding of syndecan‐1 ectodomain with its chondroitin sulfate chains intact but with its heparan sulfate chains largely removed (Figure 2c).
5. The dimeric syndecan‐1 ectodomain then is left with at least four chondroitin sulfate chains making it able to bind to infected RBCs in the intervillous space and, since it is effectively multivalent, to link infected RBCs together causing them to aggregate (Figure 2d). It may be that *P. falciparum* exploits syndecan‐1 shedding to enhance virulence as was reported for *Pseudomonas aeruginosa* (Park et al., 2001).
6. The presence of fibronectin II‐like repeats in MMP‐9, the binding of fibrinogen and fibrin to fibronectin, and the resultant binding of fibrin to MMP‐9 could allow fibronectin and MMP‐9 to contribute to the formation of the intervillous mesh.
7. The similar incidences of intervillous fibrin between gestational Weeks 23 and 27 in a study or pregnant women in the United States (24%) and the estimate provided here for the prevalence of placental malaria in sub‐Saharan Africa (30%), a high transmission area, could be more than a coincidence. It seems possible that the fibrin that occurs spontaneously in the placentas of some women provides the initial lattice for the trapping of aggregated infected RBCs (that may also have platelets and uninfected RBCs bound to them) and leads to placental malaria. Thus, the presence of intervillous fibrin mesh could predispose women to placental malaria.
8. The finding of pigment or cells (without parasites) within perivillous fibrinoids on the surface of the syncytiotrophoblast in cases of past placental malaria could indicate that these fibrinoids are the product of the intervillous mesh after it has collapsed.

## 9 POSSIBLE IMPLICATIONS FOR THE TREATMENT OF PLACENTAL MALARIA

If intervillous fibrin before infection is a prerequisite or contributing factor for placental malaria, then treatments that eliminate intervillous fibrin could be preventative for placental malaria. Also, agents that inhibit heparanase or the shedding of syndecan‐1 ectodomain (in particular, MMP‐9) could be effective against placental malaria.

Doxycycline is a tetracycline antibiotic that has been used to treat and prevent malaria (Cross, Ling, Day, McGready, & Paris, 2016; Gaillard, Madamet, & Pradines, 2015; Tan et al., 2011; WHO, 2015) and has been recommended for intermittent preventive treatment of *P. falciparum* malaria in pregnant women (Gaillard, Boxberger, Madamet, & Pradines, 2018). Doxycycline is also an inhibitor of MMP‐9 (Dursun, Kim, Solomon, & Pflugfelder, 2001; Sochor et al., 2009) and can be used for treatment of conditions related to its MMP‐9 inhibitory activity, for example, chronic wounds (Stechmiller, Cowan, & Schultz, 2010) and acute lung injury (Doroszko et al., 2010; Fujita et al., 2007; Zhang, Gong, Liu, Guo, & Ge, 2014). Doxycycline can also inhibit the pro‐inflammatory effects of IL‐17 (Obradović et al., 2016). Tetracyclines share a linear fused tetracyclic nucleus and some tetracyclines (including the original tetracycline, referred to as tetracycline) not including doxycycline have been shown to cause dental staining in human neonates when administered during the second or third trimesters (Cohlan, 1977; Kline, Blattner, & Lunin, 1964; Toaff & Ravid, 1966). Initially contraindicated for use in pregnancy because of a possible tetracycline class effect, recent reviews have found little evidence to suggest an adverse effect of doxycycline on pregnancy (Cross et al., 2016; Gaillard et al., 2015) and doxycycline use during the first and second trimesters to combat various infections other than malaria has had positive effects on pregnancy outcome (Gaillard et al., 2018; Kazy, Puho, & Czeizel, 2007). Doxycycline may have beneficial effects on placental malaria due to both its antibiotic effects and its activity as an MMP‐9 inhibitor and thus may be particularly useful against placental malaria.
